# Position-specific prediction of methylation sites from sequence conservation based on information theory

**DOI:** 10.1038/srep12403

**Published:** 2015-07-23

**Authors:** Yinan Shi, Yanzhi Guo, Yayun Hu, Menglong Li

**Affiliations:** 1 College of Chemistry, Sichuan University, Chengdu, Sichuan 610064, PR China

## Abstract

Protein methylation plays vital roles in many biological processes and has been implicated in various human diseases. To fully understand the mechanisms underlying methylation for use in drug design and work in methylation-related diseases, an initial but crucial step is to identify methylation sites. The use of high-throughput bioinformatics methods has become imperative to predict methylation sites. In this study, we developed a novel method that is based only on sequence conservation to predict protein methylation sites. Conservation difference profiles between methylated and non-methylated peptides were constructed by the information entropy (IE) in a wider neighbor interval around the methylation sites that fully incorporated all of the environmental information. Then, the distinctive neighbor residues were identified by the importance scores of information gain (IG). The most representative model was constructed by support vector machine (SVM) for Arginine and Lysine methylation, respectively. This model yielded a promising result on both the benchmark dataset and independent test set. The model was used to screen the entire human proteome, and many unknown substrates were identified. These results indicate that our method can serve as a useful supplement to elucidate the mechanism of protein methylation and facilitate hypothesis-driven experimental design and validation.

During biosynthesis, the polypeptide chains that are created by ribosomes undergo a series of “product-forming” steps, such as cutting, folding and posttranslational modification (PTM). PTMs provide the proteome with structural and functional diversity. One of the most important and reversible PTMs, methylation, was discovered more than 40 years ago^1^ and predominantly occurs on arginine (Arg) and lysine (Lys) residues. Protein methylation is involved in the regulation of a wide range of biological processes, which include transcriptional regulation, signal transduction and regulation of gene expression^2^,^3^,^4^. Unsurprisingly, Arg-methylation, Lys-methylation and the associated regulatory enzymes are implicated in various human diseases^5^,^6^,^7^. Understanding the mechanisms of protein methylation is crucial for the dynamic proteome analysis of these diseases, and represents a very attractive target for drug discovery to prevent their onset^8^.

Identifying methylation sites is an essential first step towards elucidating the mechanism of protein methylation. However, it is laborious, time-consuming and usually expensive to identify protein methylation sites by using conventional experimental techniques such as mass spectrometry, methylation-specific antibodies, and CHIP-CHIP analysis^9^,^10^,^11^. The next generation of computational methods thus needs to address the above drawbacks to enable the efficient prediction of potential methylation sites in protein sequences. Considerable effort has been made in this regard to predict methylarginine and methyllysine sites. Plewczynski *et al.*^12^ built the first methylation site predictor, named AutoMotif, by using the regular expression technique. Daily *et al.*^13^ used support vector machine (SVM) to develop a method based on the hypothesis that PTMs preferentially occur in intrinsically disordered regions. Chen *et al.*^14^ designed the webserver MeMo, which utilized an orthogonal binary coding scheme to represent sequence fragments. Shao *et al.*^15^ developed a predictor called BPB-PPMS, which combined bi-profile Bayes feature extraction with SVM. Further, Shien *et al.*^16^ proposed MASA, which considers both sequence information and structural characteristics such as the accessible surface area (ASA) and the secondary structure of residues surrounding methylation sites. Hu *et al.*^17^ presented a method based on multi-sequence features and a nearest neighbor algorithm. Li *et al.*^18^ developed Methy_SVMIACO, which is based on SVM and improved ant colony optimization algorithm (IACO). Later, another predictor, PMeS constructed by Shi *et al.*^19^, improved predictions based on an enhanced feature scheme and SVM. Shi *et al.*^20^ also presented a method called PLMLA that incorporated protein sequence information, secondary structure and amino acid properties to predict methyllysine sites. Xu *et al.*^21^ proposed a predictor based on the conditional random field (CRF). CkASSP was built using the composition of K-spaced amino acid pairs by Zhang *et al.*^22^ Recently, Lee *et al.*^23^ constructed two SVM models by using lysine-methylation data from histone and non-histone proteins to predict methyllysine sites. Qiu *et al.*^24^ developed a method called iMethyl-PseAAC by incorporating physicochemical, sequence evolutionary, and structural information into a pseudo amino composition analysis. However, most of these methods applied an orthogonal encoding scheme to characterize the peptide sequence information such that each amino acid is always represented by the same 20-bit binary vector, regardless of where it occurs. It is obvious that an orthogonal encoding scheme cannot contain positional preferences for amino acids. Moreover, although satisfactory prediction results have been achieved by previous studies, the datasets used in these studies always contain redundant sequences; thus, their reported performance is probably overly-optimistic.

Given these common drawbacks to the available methods, a novel effective method was developed from sequence conservation alone using information theory to predict methylarginine and methyllysine sites. We propose position-specific conservation difference profiles by information entropy (IE) to encode peptide sequence, which can fully reflect the position-specific amino acid distribution differences between methylated and non-methylated peptides. We also attempted to identify distinctive positions (optimal position subset) from the far sides of a longer peptide sequence by combining position ranking via IG and stepwise position selection via SVM. Finally, two SVM models were built to predict methylarginine and methyllysine sites, respectively, using the position-specific conservation difference profiles of the corresponding optimal positions and a non-redundant dataset. Our method yields more promising predictions based on comparisons with other two existing methods using common independent tests. Moreover, proteome-wide predictions provide a valuable resource for the experimental validation of new methylation sites and for the generation of useful hypotheses. The source code, datasets and SVM models can be freely found at http://cic.scu.edu.cn/bioinformatics/SourceCode_and_SVMmodel.zip.

## Result and Discussion

### Difference analysis of position-specific conservation of amino acids

The distribution of amino acid residues is the basic information of a protein sequence. Traditionally, an orthogonal binary encoding scheme was used to extract the compositional characteristics of amino acid sequences. It ranks the 20 amino acids as ACDEFGHIKLMNPQRSTVWY. Each amino acid is represented by a 20-bit binary vector. For example, alanine, A, is converted to 10000000000000000000, and cysteine, C, is 01000000000000000000, and so on. The final dimension of the feature vector is 20*N, where N is the sequence length.

**Figure 1:**
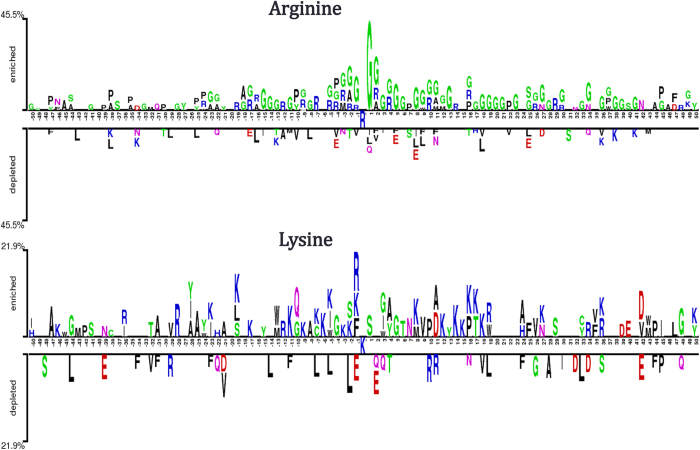
A two sample logo to show position-specific distribution difference of amino acid residues between methylated and non-methylated peptides for arginine and lysine methylation, respectively.

**Figure 2:**
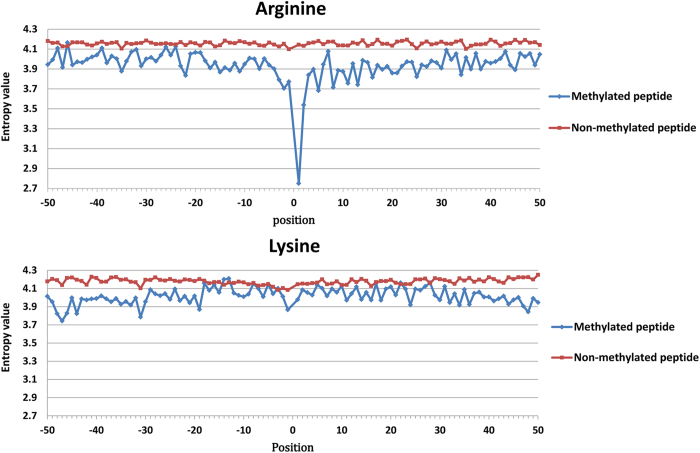
Comparison of conservation in each position between methylated and non-methylated peptides through Information entropy value for arginine and lysine methylation, respectively.

The above-mentioned representation is a position-independent matrix. In other words, a particular residue always receives the same encoding vector, regardless of the positions it occupies in a peptide sequence. Based on two sequence datasets in the benchmark for arginine and lysine, respectively, a Two Sample Logo^25^ was adapted to generate the graphical sequence logo (p < 0.05 by t-test) that can detect and display statistically significant differences in position-specific amino acid compositions between methylated and non-methylated datasets after multiple sequence alignments. As shown in Fig. 1, the distributions of the same residue have a detectable difference in flanking positions based on parallel contrast. As described by Ding *et al.*^26^, from a biological perspective, the same residue may contain different information throughout a protein’s evolutionary history, and a residue in a conserved position usually has stronger functional relevance than does a residue in a non-conserved position. Moreover, we observed that the distributions of amino acid residues are quite different between methylated and non-methylated peptides at each position, based on the vertical contrast shown in Fig. 1. Considering that the imbalanced data between positive and negative samples might give rise to the difference, we treated a balanced negative sub-sample as dummy positive samples to draw the Two Sample Logo. As shown in Supplementary Fig. S1, almost no differences were observecd. Thus it can be seen that the main difference between methylated peptides and non-methylated peptides is due to the reason that residues in the flanking positions of methylation sites are more conservative with higher specificity than those of non-methylation sites, rather than the limited positive samples. As more validated methylated sites from high-throughput proteomic experiments become available, a more statistical difference analysis can be expected. Figure 2 shows the comparison of the conservation score of each position between methylated and non-methylated peptides based on IE. This comparison further demonstrates that residues in the flanking positions of methylation sites are more conserved and have a higher specificity than do those of non-methylated sites. For example, glycine (G) occurs with the highest frequency in the +1 position in Arg-methylated peptides, as shown in Fig. 1. As a consequence, the +1 position has the lowest entropy value (Fig. 2), which indicates that it is the most conserved position. Indeed, motif analysis has revealed that many Arg-methylation sites are associated with RGG/RXG/RGX^27^ or GXXR^13^ motifs. In contrast, no amino acids surrounding methylated lysines are obviously conserved in the currently available data. However, we still did find some positions where there are relatively stronger differences in conservation, such as the −1, −19, 14 and 30 positions (Fig. 2).

Based on the above analysis, we constructed position-specific conservation difference profiles to replace the orthogonal binary encoding scheme. Peptide sequence information was characterized using the position-specific conservation difference profiles, which reflect not only the position-specific compositions of each amino acid but also the different distributions of amino acids between methylated and non-methylated peptides. Overall, the difference in conservation of amino acid between methylated and non-methylated peptides can be captured by IE and that the differences can be sufficiently obvious to differentiate them. Therefore, based only on the conservation profiles of peptides developed by IE, a good prediction of methylation sites can be expected.

### Selection of distinctive positions

All positions in a protein are not equally important. Some are essential for the proper structure and function of the protein, whereas others can be readily replaced^28^. For a given sequence fragment, conservation varies from one position to another, and some residues near methylation sites might provide a minimal contribution to their identification, whereas some positions far away from the target site in the sequence might be close to the site in the structure. Supplementary Fig. S2 shows that for arginine, the position [−39, −50] and [25, 29] are close to the methylation site in 3D structure of P06746_83. For Lysine, the position [−25, −35] and [28, 35] are close to the methylation site in the 3D structure of Q01853_315. Thus, it is necessary to identify the positions that contribute to the methylation of a given residue in the optimal position subset as opposed to those in the initial window. Previous studies on methylation prediction only focused on the closest positions surrounding the methylation sites, with at most 7 residues symmetrically upstream and downstream of the methylated residue. Our study attempted to find important positions that are far from the target site in the primary sequence.

**Figure 3:**
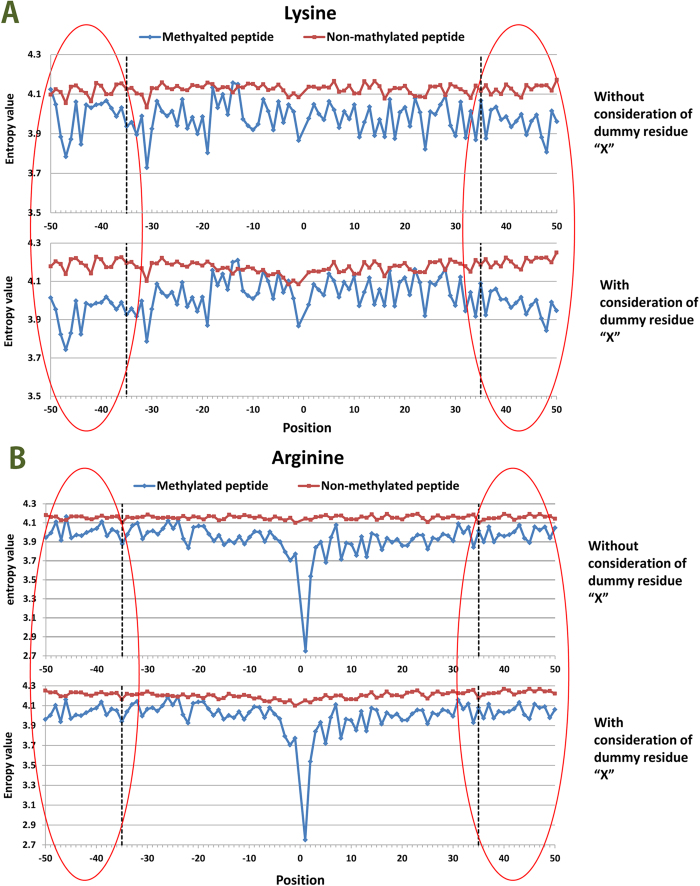
Comparison of difference in conservation between methylated and non-methylated peptides based on the algorithm (IE) with consideration of dummy residue “X” and without consideration of the residue “X”.

Therefore, a wide range of methylated peptide lengths were considered, with at most 101 residues from position −50 to 50. For the terminal methylation sites, dummy residues were represented as ‘X’ and placed in the vacant positions to complete the window in the longest methylated peptides. For the Lys-methylation dataset, statistical analysis shows that only 31% peptides have lengths of 101 without a dummy residue. However, 65% of all of the peptides did not include “X” when the initial window size was set at 71. Because the residue “X” is of no biological significance, the difference in conservation between methylated and non-methylated peptides might be strengthened by these dummy residues. As shown in Fig. 3A, compared with the difference in conservation based on the algorithm without considering “X” residues , an obvious increase in the difference in conservation was found when “X” residues were considered, especially given a window of 15 residues on both sides. At the interval of [−35, 35], the difference in conservation is nearly identical, regardless of whether “X” is included. In contrast, in the Arg-methylation dataset, peptides with a length of 101 residues that do not include the dummy residue “X” account for 60% of the dataset. Figure 3B shows that there is only a tiny variation in the difference in conservation between methylated and non-methylated peptides, considering dummy residue “X”. The minor effects of “X” on both ends of peptides can be neglected. Therefore, initial window sizes of 101 and 71 were used to analyze Arg-methylation and Lys-methylation, respectively.

**Figure 4:**
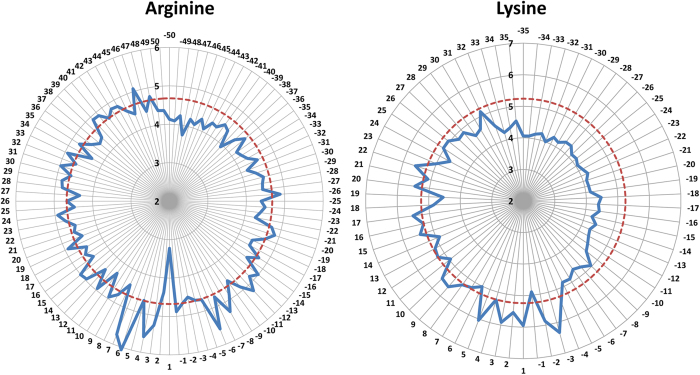
Relative importance of the different positions. The radar chart represents the −log_2_ ratio of information gain among each position. The broken circle in red represents threshold and positions inside the broken circle are the optimal position subset.

IG was used to measure the importance of positions on both sides of the methylation sites. IG scores were obtained for these positions based on the benchmark dataset, and they are summarized in Supplementary Table S1. The positions were sorted from high to low IG scores. As observed in Fig. 4, some positions that are far from the methylation site have high IG scores, whereas some closer positions have lower scores. The overall trend was that the scores of the upstream positions were generally higher than those of downstream positions, which implies that methyltransferases tend to bind to residues upstream of methylation sites. This finding is consistent with the result of Lu’s work^29^ on the prediction of lysine acetylation sites.

**Figure 5:**
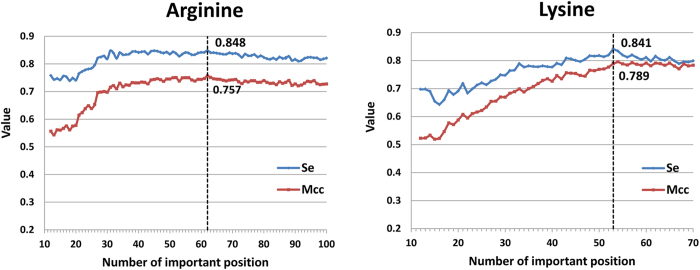
The way of selecting distinctive positions combines IG position ranking and stepwise position selection via SVM for arginine and lysine methylation, respectively. The broken line represents the optimum on the basis of Se an MCC.

We chose relatively important positions as the optimal position subset, and those with IG scores higher than the threshold were used to rebuild new methylation peptides. To identify distinct positions, stepwise position selection based on SVM modeling was implemented. At each round of stepwise position selection, the last position from the IG-ranked position list was deleted, and the 10 SVM models on the remaining top positions were constructed because there were 10 training sets for Lys-methylation or Arg-methylation. The stepwise position selection was repeated 89 times until the number of neighboring positions decreased from 100 to 12 for Arg-methylation and 59 times until the number of the neighboring positions decreased from 70 to 12 for Lys-methylation. During this process, the average prediction performance of 10 SVM models on each position subset was referenced to select the optimal one. As observed in Fig. 5, by iteratively deleting positions from the initial window, the sensitivity (Se) and Matthew’s correlation coefficient (MCC) of the model increased moderately and then declined relatively rapidly. The reason for these trends may be that when the position subset was too large, redundant information was included, whereas the inclusion of too few neighboring positions led to the loss of useful information.

The two-step method of selecting distinctive positions combines IG position ranking and stepwise position selection via SVM and provides a practical approach for selecting the relatively important positions, which is similar to the feature selection method described by Li *et al.*^30^. In Fig. 5, we can see that for Arg-methylation, the model based on the optimal position subset containing 62 positions from the initial 101 positions yields the highest Se and MCC, and that the optimal position subset containing 53 positions from 71 ones gives the best performance for Lys-methylation. The relative importance of the positions and the final threshold are shown in Fig. 4. The radar chart represents the −log_2_ ratio of information gain among each position. The broken circle in red represents the IG score threshold, and the positions inside the broken circle are the important for the identification of methylation sites.

### Determination of the best initial window size

**Figure 6:**
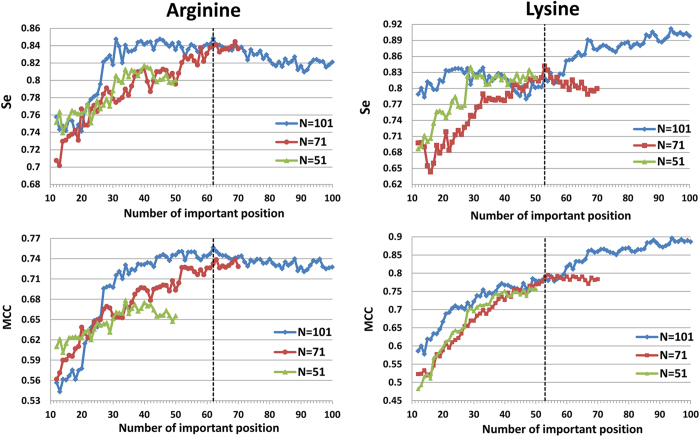
Determination of the best initial window size for arginine and lysine methylation, respectively. The broken line represents the optimum on the basis of Se and MCC, respectively.

The above distinct positions were selected based on an initial window size of 100 and 70 for Arg-methylation and Lys-methylation, respectively. Here, we further verified the effects of different initial window sizes on the prediction performance by comparing the results using different initial window sizes. The resulting performance (Se and MCC) of models combining IG position ranking and stepwise position selection via SVM using different initial window sizes is illustrated in Fig. 6. For arginine methylation, the Se and MCC gradually increased when the initial window size increased from 51 to 71 and then to 101. The optimal initial window size for Arginine methylation is 101. For lysine methylation, the predictive performance also gradually increased when the initial window size increased from 51 to 71 and then to 101. However, the high sensitivity of the model using 101 positions was due to the inclusion of the dummy residue “X” as previously described. Although we replaced the residue “X” with 0 in the encoding process to avoid its effect, pattern recognition can still identify such signals. Therefore, we selected 71 as the optimal window size.

Through our encoding scheme, a feature vector of a peptide with N variables was obtained. N is the number of distinctive positions, and each variable is represented by the conservation profile. We created a 62-dimensional feature vector for the Arg-methylation dataset and a 53-dimensional one for the Lys-methylation dataset.

### Prediction performance based on benchmark datasets and selection of the representative model

As described in Data Collection and Preprocessing, we trained 10 models based on the final optimal position subset for Arg-methylation and Lys-methylation separately. The prediction performance in the case of 5-fold cross-validation is given in Table 1. For Arg-methylation, the average Se, specificity (Sp), accuracy (Acc), and MCC of the 10 prediction models are 84.83%, 90.36%, 87.60%, and 0.76, respectively. For Lys-methylation, the average values are 84.14%, 94.09%, 89.12%, and 0.79, respectively. The ROC curves of all 10 models on 5-fold cross-validation are given in Supplementary Fig. S3.

To determine whether the predictive model is over-fitting for the benchmark datasets, we re-clustered the benchmark datasets using 40% identity threshold with CD-HIT webserver. A total of 99 positive samples and 526 negative samples were obtained for Arg-methylation, and 94 positive samples and 436 negative samples were for Lys-methylation. Four-fifths of these samples were used as training set, and the rest served as the testing set. As usual, the balanced negative training set was randomly selected from all negative training samples, and the procedure was iterated 10 times. For each paired positive and negative training set, a model was built by 5-fold cross-validation. Moreover, each model was selected to evaluate the testing set. The results are listed in Supplementary Tables S2 and S3. The cross-validation results (Table S2) indicate that the models on the 40% sequence identity dataset yielded an average Se, Sp, Acc, and MCC of 81.59%, 86.73%, 84.16% and 0.69 for Arg-methylation and 84.57%, 85.84%, 85.20% and 0.71 for Lys-methylation, respectively. The performance of the model on the 40% identity training dataset is equivalent to that of the model on the 70% identity benchmark dataset. We can thus infer that the model cannot be over-fitted due to the relatively high sequence identity of the original benchmark dataset. Because the peptides in the test set share lower than 40% identity with those in the training dataset, the model on the test set (Table S3) also give a high average Se of 84% for Arg-methylation and Lys-methylation, which indicates our method is robust.

**Figure 7:**
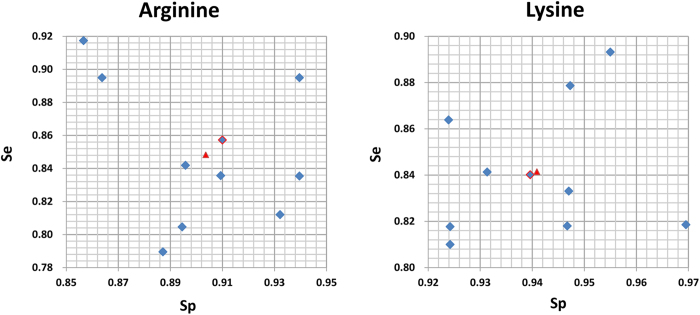
Selection of the representative model for arginine and lysine methylation, respectively. The red triangle represents the average performance of all 10 models. The model with a red border represents the representative model.

**Figure 8:**
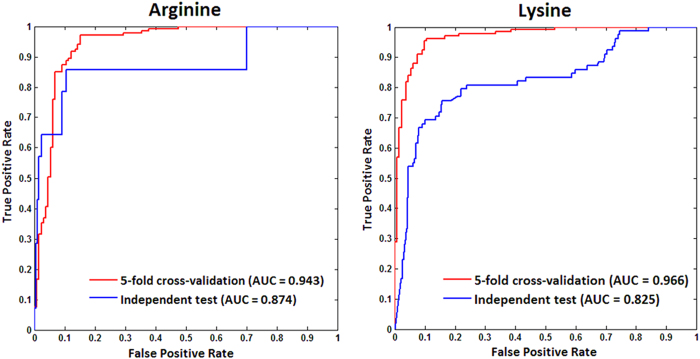
ROC curves of 5-fold cross-validation and independent test on the representative model for arginine and lysine methylation, respectively.

A final model must be selected as the representative model to predict new methylation sites. Because the negative training set was randomly selected from the original large number of negative samples, the final best negative training set in the representative model would have the characteristics of the overall negative samples. We mapped the performance of each model to the square chart, where Sp was the x-axis and Se was the y-axis, as shown in Fig. 7. Among the 10 models, we selected the one that gave gives the nearest performance to the average as the representative final model. In the end, the models with a red border in Fig. 7 were selected as the representative models for Arg-methylation and Lys-methylation. Furthermore, the receiver operating characteristic (ROC) curves of the representative models for Arg-methylation and Lys-methylation are shown in Fig. 8, and the corresponding values of the area under the curve (AUC) are as high as 0.943 and 0.966, respectively.

### Comparison with existing predictors based on common independent test datasets

To further validate and assess our method against other sources of methylation data, we compared our method using the representative models with other two existing predictors, iMethyl-PseAAC^24^ and BPB-PPMS^15^, which are available via web servers and are based on common independent test datasets. From the UniProtKB/Swiss-Prot database (version 2014_08), we obtained 12 proteins containing 14 experimental methylarginine sites and 210 non-methylarginine sites, and 44 proteins containing 78 experimental methyllysine sites and 611 non-methyllysine sites, as common independent test datasets. None of these proteins are included in our benchmark datasets. To avoid the memory effect or bias in favor of the other two predictors, neither of the samples in our independent datasets occurs in the datasets used to train the other two predictors. Table 2 clearly shows that our method yielded promising performance for both types of methylation sites. For Arg-methylation, the other two predictors achieved high specificity, but their sensitivity was relatively low, and our method yielded much higher sensitivity. For Lys-methylation, the relatively low specificity of 84.45% for methyllysine site prediction obtained by our method indicates that approximately 95 non-methyllysine sites were predicted to be potential methyllysine sites. It is possible that a large number of unknown arginine or lysine methylation sites will be discovered in the future. These results suggest that our method is equivalent to other predictive systems based on the current available testing data. Moreover, Fig. 8 also presents the ROC curves of independent tests on the representative models. For Arg-methylation and Lys-methylation, the AUC values are 0.874 and 0.825, respectively, which further indicates the promising practical performance of our method. We also implemented sequence alignment between the common independent test dataset and our benchmark dataset using BLAST with a 30% sequence similarity threshold. A subset was remained covering 8 experimental methylarginine sites and 99 non-methylarginine sites; 34 experimental methyllysine sites and 210 non-methyllysine sites. The performance of our method on this sub-independent dataset is shown in Supplementary Table S4. Our method gives a high accuracy of 92% and 83% for Arg-methylation and Lys-methylation, respectively, on such a sub-testing set sharing low sequence identity with the benchmark set, which further verified the generalizability of our method.

### Human proteome-wide prediction by our method

To facilitate the high-throughput *in silico* prediction of methylation sites on a proteomic scale, we used our method to screen the complete human proteome of all 14260 proteins. They exist at protein level and have not been annotated by any methylation information from the UniProtKB/Swiss-Prot database (version 2014_08). A total of 483855 arginine peptides and 473975 lysine peptides were finally obtained. Our high-confidence analysis identified 44423 predicted methylarginine sites and 57712 predicted methyllysine sites. The proteome-wide predictions represent a valuable resource for the experimental validation of novel methylation substrates and generation of useful hypotheses.

## Conclusion

Here, we have developed a simple but effective method for predicting protein methylation sites that is based only on sequence conservation. Rigorous model construction and testing was implemented. First, an unbiased benchmark dataset was constructed to establish statistical predictors by sequence alignment of the peptides with sequence identity of 70%. Second, we proposed position-specific conservation difference profiles based on IE to characterize peptide sequence information, which reflects not only the position-specific composition of the amino acids, namely the position preference of amino acids, but also the different distributions of amino acids in each position by comparing methylated and non-methylated peptides. Third, by combining position ranking via IG and stepwise position selection via SVM, distinct positions were identified in a wider range of neighboring residues around the methylation sites, which overcame the potential that overly short peptides would lead to a loss of other neighboring information and redundant information would be introduced by increasing the length of the peptides. Finally, to avoid erroneous performance of the prediction model, the selection procedure for negative samples and the construction of model construction was iterated 10 times. The final representative model was selected because it had the closest performance to the average among the 10 models. This method achieved promising performance. The proteome-wide predictions represent a valuable resource for the experimental validation of novel methylation substrates. We expect that our method will provide powerful guidance for hypothesis-driven experimental studies of new methylation sites.

## Methods

### Data Collection and Preprocessing

All training data for arginine and lysine methylation sites were derived from UniProtKB/Swiss-Prot database (version 2014_08). A total of 117 proteins covering 294 experimentally verified methylarginine sites and 227 proteins covering 493 experimental methyllysine sites were obtained by searching for “Omega-N-methylarginine,” “Omega-N-methylated arginine,” “symmetric dimethylarginine,” “asymmetric dimethylarginine” and “dimethylated arginine” for methylarginine and “N6,N6-dimethyllysine,” “ N6,N6,N6-trimethyllysine,” “N6-methyllysine” and “N6-methylated lysine” for methyllysine in the field “PTM/Processing” with experimental verification. Those marked as “probable,” “possible” or “by similarity” were excluded. Non-methylated sites for arginine and lysine include the remaining arginine and lysine residues that were not annotated with any methylation information on the proteins containing experimentally validated methylated sites. Although not all of these non-methylated sites are confirmed true negatives, it is reasonable to believe that the large majority of them are^31^.

For both the positive and negative datasets, an initial window with each arginine or lysine in the middle surrounded by flanking residues was defined. In practice, the initial window size was set to N = 2*n* + 1 (*n* = 25, 35, 50), where *n* is the number of upstream or downstream residues, and a peptide sequence was extracted from protein sequence as^32^


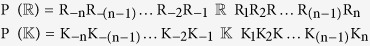


For terminal methylation sites, where the number of flanking residues was less then *n*, an equal number of dummy residue ‘X’ was added to complete the window.

Because homologous sequences could lead to an overestimation of the prediction performance, considering the limited number of experimentally verified methylation sites, we clustered peptide sequences at the 70% identity level using the CD-HIT webserver^33^ for initial window sizes of 51, 71, and 101. Moreover, we excluded samples that were self-conflicting, namely, those that occurred in both methylated and non-methylated peptides. Finally, we used non-redundant positive and negative peptide samples as the benchmark datasets for Arg-methylation and Lys-methylation. Table 3 shows the statistics of the benchmark datasets.

To perform the cross-validation, all of the non-redundant positive samples were selected as the positive training set. The balanced negative training set was randomly selected from all non-redundant negative samples. However, the randomly selected negative training set might not sufficiently encompass the characteristics of the overall non-redundant negative samples. Therefore, to ensure unbiased and objective results, the selected procedure was iterated 10 times. For each paired positive and negative training set, a model was built, and 5-fold cross-validation was carried out. The average predictive performance of the ten models was calculated.

### Information Theory

#### Feature extraction-Information Entropy (IE)

Information entropy (IE) proposed by Shannon^34^ has been considered the most important concept in the information theory. Shannon entropy is the expected value of the uncertainty for a given random variable. High uncertainty can contain more information, so it is defined as a quantified factor to measure the information content^35^.

Shannon entropy is one of the simplest and most common measures of conservation at a site in a protein sequence in the field of bioinformatics^36^. It is defined for a position/column *c* as


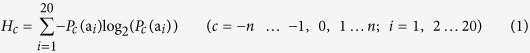


where *n* is the number of upstream or downstream residues, {*a*_*i*_} is the 20 element set of amino acids, and *P*_*c*_ (*a*_*i*_) is the frequency of the *i*th amino acid occurring in position *c* in a multiple sequence alignment. The resulting value varies from 0, in the case of complete conservation, to 4.32, which occurs when all 20 residues are equally distributed.

In this study, we proposed position-specific conservation difference profiles according to the information content of every residue in the peptide sequences in benchmark datasets. We first counted the amino acid frequency of both methylated and non-methylated samples after sequence alignment. The information content of each amino acid at each position can be calculated in the positive and negative datasets, respectively, as follows:


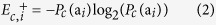



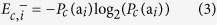


The position-specific conservation difference profiles (*E*_*c,i*_) with the dimension of 21*N (where 21 represents the 20 element set of amino acids and dummy residue ‘X’) was generated through formula (4). For the dummy residue ‘X,’ ‘0’ was used to represent it numerically regardless of its position. The two profiles for Arg-methylation and Lys-methylation are presented in Supplementary Table S5.


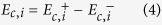


Through position-specific conservation difference profiles, each peptide sequence can be encoded by vector 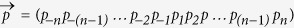, and the dimension is same as the window size.

#### Position /feature selection-Information Gain (IG)

Information gain (IG), which measures the decrease in information entropy when a given variable is used to group values of another variable, can also be considered a measure of the degree of ordering^37^. Here, we employed information gain (IG) as a feature selection method^38^ to distinguish the importance of different positions for methylated peptides relative to non-methylated peptides. The IG scores on different positions were calculated through the following procedure:

The entropy of a variable *X* is defined as


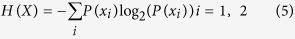


where {*x*_i_} is the positive sample or negative sample and *P*(*x*_i_) is the prior probability of methylated and non-methylated sites in benchmark samples.

The conditional entropy of *X*, given another variable *Y* (when IG scores of positions are extracted, the amino acid type is determined) is defined as


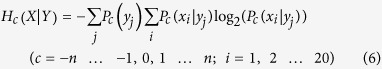


where *n* is the number of upstream or downstream residues, {*y*_*i*_} is the 20 element set of amino acids, *P*_*c*_(*y*_j_) is the frequency of the *j*th amino acid occurring in position *c* in benchmark samples, and *P*_*c*_(*x*_i_ | *y*_j_) is the posterior probability of X given the value *y*_*j*_of *Y*.

The amount by which the entropy of *X* decreases reflects additional information about *X* provided by *Y* and is called information gain^39^


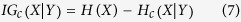


According to this measure, *Y* has a stronger correlation with *X* than it does with *Z* if IG(*X|Y*) > IG(*Z*|*Y*). The larger the IG score of a position is, the more important the position is, which indicates that the position has a greater influence on the methylation site. Using this method, we obtained the ranking list of the IG scores of all positions.

#### SVM learning and Performance evaluation

As a machine-learning method of binary classification, SVM maps the input datasets into a higher dimensional space and aims to create a hyper-plane that can separate the data into two classes with the maximum margin. SVM is considered to be one of the most accurate machine-learning algorithms and has been frequently used to address classification problems in bioinformatics, such as secondary structure prediction^40^, protein fold recognition^41^ and protein-protein interaction^42^.

In our study, SVM was used to identify whether the residue was a methylation site. For the actual implementation, the LIBSVM package^43^ was used, and a radical basis function (RBF) was chosen as the kernel function. To maximize the performance of the SVM algorithm, the optimal set of parameters, the penalty parameter *C* and the kernel width parameter *γ*, were obtained by the grid search method in LIBSVM.

To train and construct a SVM model, 5-fold cross-validation tests were performed. The training dataset is randomly divided into five groups. Each group in turn is used as a testing set, and the remaining four groups are merged to train the SVM model. The average performance of 5 repetitions is the final result of the 5-fold cross-validation tests. Five effective performance metrics, i.e., sensitivity (Se), specificity (Sp), accuracy (Acc), Matthew’s correlation coefficient (MCC) and AUC, were used for performance assessment. The AUC is the area under the receiver-operating characteristic (ROC) curve and is presented as a plot of the true positive rate against the false positive rate. An AUC value of 1.0 indicates perfectly accurate prediction, whereas 0.5 signifies completely random prediction. Se, Sp, and Acc indicate the predictive success rates on positive, negative, and overall samples, respectively. MCC accounts for true and false positives and negatives and is usually considered a balanced measure that can be used even if the classes are of very different sizes. These metrics are defined as follows:


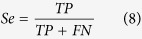



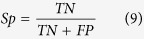



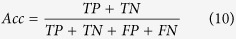



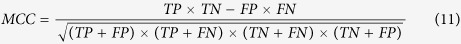


where TP, FP, TN, and FN are the numbers of true positive, false positive, true negative, and false negative samples, respectively.

## Additional Information

**How to cite this article**: Shi, Y. *et al.* Position-specific prediction of methylation sites from sequence conservation based on information theory. *Sci. Rep.* **5**, 12403; doi: 10.1038/srep12403 (2015).

## Supplementary Material

Supplementary Information

## Figures and Tables

**Table 1:** The performance of 10 models in 5-fold cross-validation on benchmark datasets for arginine and lysine methylation.

Training Set	Model	Se (%)	Sp (%)	Acc (%)	MCC
Arginine	1	80.46	89.46	84.96	0.70
	2	83.53	93.96	88.75	0.78
	3	81.20	93.22	87.21	0.75
	4	89.49	86.38	87.93	0.76
	5	84.19	89.59	87.24	0.75
	6	89.49	93.96	91.72	0.84
	7	78.95	88.72	83.83	0.68
	8	83.56	90.94	87.25	0.75
	9	91.74	85.67	88.70	0.78
	10^*^	85.73	91.00	88.36	0.77
	Average	84.83	90.36	87.60	0.76
Lysine	1	81.00	92.42	86.71	0.74
	2	81.85	96.95	89.40	0.80
	3	84.13	93.13	88.63	0.78
	4	81.79	94.67	88.23	0.78
	5	86.38	92.39	89.39	0.79
	6^*^	84.02	93.96	88.99	0.79
	7	87.86	94.73	91.30	0.83
	8	89.32	95.50	92.41	0.85
	9	83.30	94.70	89.00	0.79
	10	81.77	92.42	87.09	0.75
	Average	84.14	94.09	89.12	0.79

**Table 2:** Performance comparison of our work with other existing predictors on common independent test datasets.

Testing Set	Method	Se (%)	Sp (%)	Acc (%)	MCC
Arginine	iMethyl-PseAAC	42.86	99.05	95.54	0.55
	BPB-PPMS	42.86	97.14	93.75	0.43
	Our Work	78.57	97.14	95.98	0.69
Lysine	iMethyl-PseAAC	96.15	58.43	62.70	0.35
	BPB-PPMS	65.38	87.56	85.05	0.43
	Our Work	74.36	84.45	83.31	0.45

**Table 3:** The statistics of the final non-redundant positive peptide samples and non-redundant negative peptide samples for arginine and lysine.

Window size	Arginine peptide		Lysine peptide	
	Methylation	Non-methylation	Methylation	Non-methylation
N = 51	156	1308	142	1175
N = 71	144	1088	132	965
N = 101	133	860	118	721
