# Supplementary material for: Position-specific prediction of methylation sites from sequence conservation based on information theory
Source: Sci Rep. 2015 Jul 23;5:12403. doi: 10.1038/srep12403 (PMC5378888; doi:10.1038/srep12403)
Supplement: Supplementary Information [file srep12403-s1.pdf]

Position-specific prediction of methylation sites from sequence conservation based on information theory

Yinan Shi, Yanzhi Guo*, Yayun Hu, Menglong Li*

College of Chemistry, Sichuan University, Chengdu, Sichuan 610064, PR China

Corresponding authors marked with *

Yanzhi Guo

Menglong Li

Tel: +86 28 85413330

Fax: +86 28 85412356

E-mail address: yzguo@scu.edu.cn

liml@scu.edu.cn

Supplementary Figures:

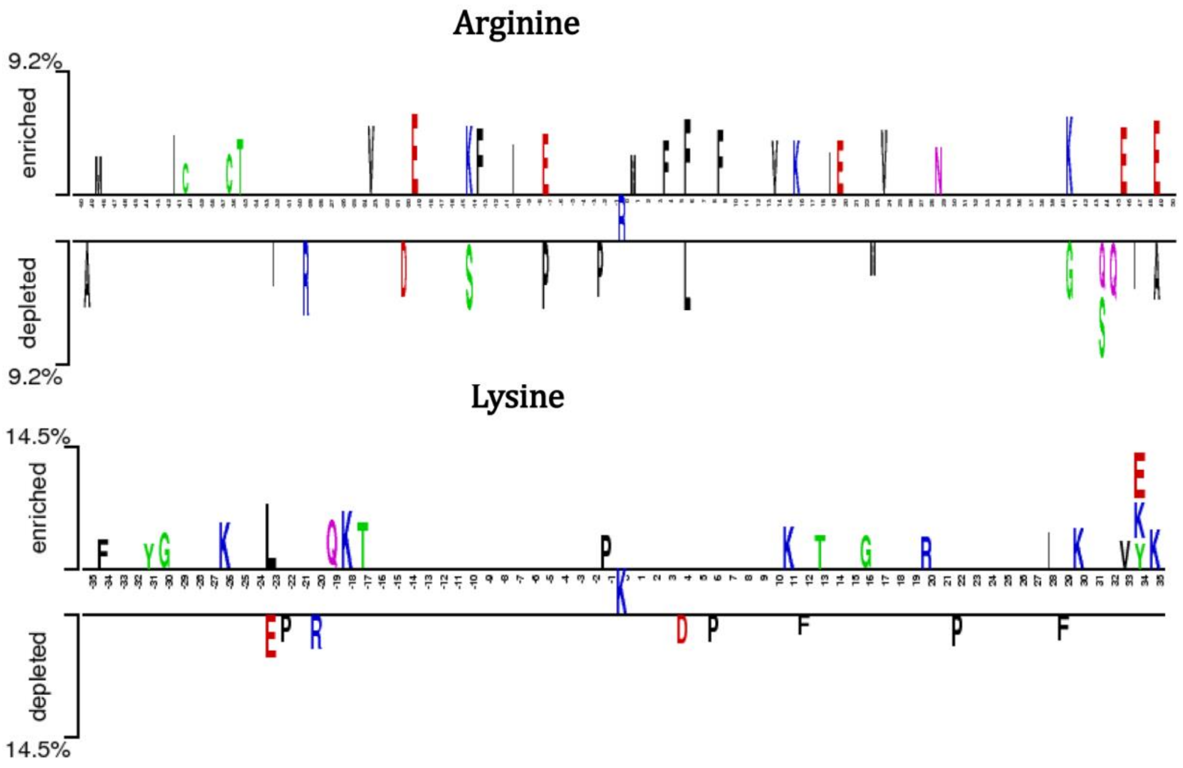

Supplementary Figure S1 | Two Sample Logo between the negative subset and the whole non-methylated samples for arginine and lysine methylation, respectively.

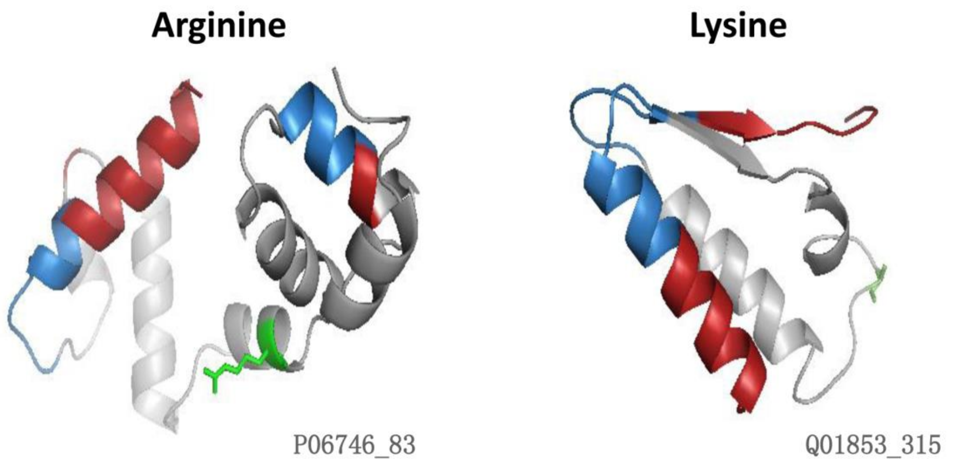

Supplementary Figure S2 | 3D-structural of methylation peptide for arginine and lysine, respectively. The amino acid in green color is the methylation site. The red ones represent that these residues far away from methylation in sequence are closer to the methylation site in structure as compared to the blue ones.

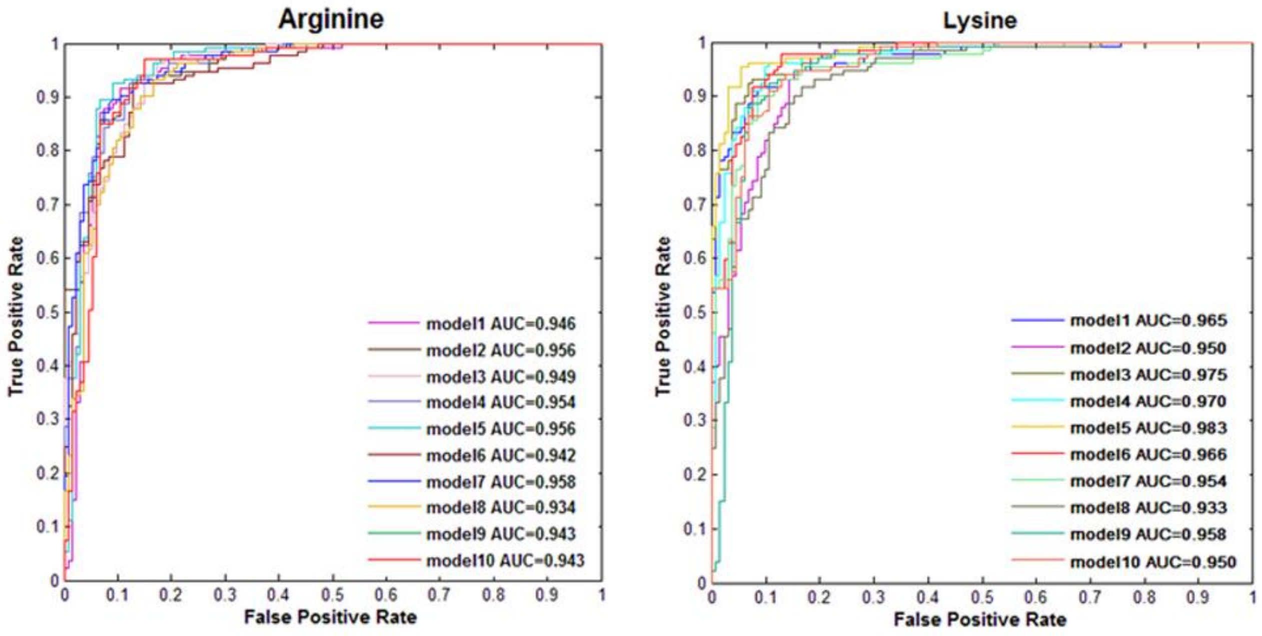

Supplementary Figure S3 | ROC curves of 5-fold cross-validation on 10 training sets for arginine and lysine methylation, respectively.

Supplementary Tables:

Supplementary Table S1| A summary of IG score of positions for arginine and lysine methylation peptide.

Positions	Arginine	Lysine
-50	0.0572	
-49	0.0586	
-48	0.0530	
-47	0.0743	
-46	0.0546	
-45	0.0586	
-44	0.0511	
-43	0.0592	
-42	0.0501	
-41	0.0513	
-40	0.0455	
-39	0.0481	
-38	0.0652	
-37	0.0496	
-36	0.0427	
-35	0.0559	0.0599
-34	0.0536	0.0598
-33	0.0504	0.0564
-32	0.0434	0.0540
-31	0.0554	0.0624
-30	0.0485	0.0531
-29	0.0434	0.0559
-28	0.0454	0.0507
-27	0.0460	0.0504
-26	0.0338	0.0573
-25	0.0478	0.0562
-24	0.0444	0.0602
-23	0.0479	0.0574
-22	0.0517	0.0536
-21	0.0372	0.0579
-20	0.0340	0.0573
-19	0.0391	0.0566
-18	0.0479	0.0450
-17	0.0481	0.0479
-16	0.0371	0.0464
-15	0.0446	0.0531

-14	0.0314	0.0468
-13	0.0345	0.0510
-12	0.0273	0.0491
-11	0.0336	0.0458
-10	0.0424	0.0427
-9	0.0334	0.0321
-8	0.0258	0.0365
-7	0.0371	0.0390
-6	0.0210	0.0339
-5	0.0387	0.0344
-4	0.0395	0.0234
-3	0.0429	0.0125
-2	0.0390	0.0171
-1	0.0339	0.0337
0	0.0000	0.0000
1	0.1069	0.0162
2	0.0480	0.0214
3	0.0262	0.0166
4	0.0208	0.0263
5	0.0394	0.0152
6	0.0149	0.0310
7	0.0189	0.0272
8	0.0471	0.0256
9	0.0365	0.0204
10	0.0323	0.0213
11	0.0501	0.0324
12	0.0324	0.0264
13	0.0461	0.0277
14	0.0321	0.0339
15	0.0312	0.0231
16	0.0458	0.0264
17	0.0415	0.0217
18	0.0455	0.0348
19	0.0331	0.0430
20	0.0459	0.0226
21	0.0410	0.0291
22	0.0432	0.0206
23	0.0370	0.0312
24	0.0328	0.0401
25	0.0448	0.0305
26	0.0392	0.0304
27	0.0493	0.0353
28	0.0356	0.0340
29	0.0347	0.0424

30	0.0400	0.0398
31	0.0318	0.0284
32	0.0407	0.0432
33	0.0328	0.0525
34	0.0507	0.0489
35	0.0366	0.0429
36	0.0441	
37	0.0490	
38	0.0467	
39	0.0334	
40	0.0338	
41	0.0394	
42	0.0350	
43	0.0356	
44	0.0416	
45	0.0461	
46	0.0297	
47	0.0470	
48	0.0366	
49	0.0482	
50	0.0487	

Supplementary Table S2 | The performance of 10 models in 5-fold cross-validation on 40% identity training set for arginine and lysine methylation.

Training Set	Model	Se (%)	Sp (%)	Acc (%)	MCC
Arginine	1	78.31	84.41	81.36	0.64
	2	80.59	80.96	80.77	0.62
	3	81.99	88.01	85.00	0.71
	4	77.13	86.69	81.91	0.65
	5	79.41	90.44	84.93	0.71
	6	83.04	88.58	85.81	0.72
	7	83.64	86.85	85.25	0.71
	8	84.43	86.64	85.54	0.71
	9	83.70	87.36	85.53	0.71
	10	83.70	87.36	85.53	0.71
	Average	81.59	86.73	84.16	0.69
Lysine	1	83.50	83.75	83.63	0.68
	2	84.83	73.50	79.17	0.59
	3	84.83	82.33	83.58	0.68
	4	84.83	89.58	87.21	0.76
	5	81.00	87.33	84.17	0.69
	6	84.67	89.25	86.96	0.74
	7	85.81	87.75	86.78	0.74
	8	85.52	88.11	86.81	0.74
	9	85.33	88.37	86.85	0.74
	10	85.33	88.37	86.85	0.74
	Average	84.57	85.84	85.20	0.71

Supplementary Table S3 | The performance of 10 models on testing set under 40% identity for arginine and lysine methylation.

Testing Set	Model	Se (%)	Sp (%)	Acc (%)	MCC
Arginine	1	81.25	87.50	86.54	0.59
	2	81.25	88.64	87.50	0.61
	3	81.25	87.50	86.54	0.59
	4	87.50	90.91	90.38	0.69
	5	87.50	87.50	87.50	0.63
	6	87.50	88.64	88.46	0.65
	7	81.25	87.50	86.54	0.59
	8	93.75	90.91	91.35	0.74
	9	81.25	89.77	88.46	0.63
	10	81.25	87.50	86.54	0.59
	Average	84.38	88.64	87.98	0.63
Lysine	1	86.67	91.78	90.91	0.72
	2	86.67	94.52	93.18	0.77
	3	86.67	86.30	86.36	0.62
	4	80.00	90.41	88.64	0.64
	5	86.67	89.04	88.64	0.67
	6	86.67	91.78	90.91	0.72
	7	86.67	90.41	89.77	0.69
	8	80.00	89.04	87.50	0.62
	9	86.67	94.52	93.18	0.77
	10	80.00	89.04	87.50	0.62
	Average	84.67	90.68	89.66	0.68

Supplementary Table S4 | The performance of our method on the subset of the common independent test set sharing <30% sequence identity with the training dataset.

Residue	Se (%)	Sp (%)	Acc (%)	MCC
Arginine	75.00 (6/8)	93.94 (93/99)	92.52	0.57
Lysine	76.47(26/34)	84.29(177/210)	83.20	0.49

Supplementary Table S5 | Position-specific distribution difference profiles for arginine and lysine methylation, respectively.

Arginine methylation

	-50	-49	-48	-47	-46	-45	-44	-43	-42	-41	-40	-39	-38	-37	-36
A	-0.019	-0.054	-0.028	-0.081	0.005	0.155	0.1079	-0.064	-0.01	0.0084	0.0501	-0.073	0.131	0.0504	-0.028
C	-0.028	0.1063	-0.064	-0.076	0.0618	-0.033	-0.055	0.0196	-0.034	-0.051	-0.003	-0.013	-0.088	0.0309	-0.109
D	-0.006	0.0986	-0.018	0.0846	0.0139	-0.057	0.0057	-0.049	-9E-04	-0.066	0.0224	0.0571	0.0249	-0.062	0.0355
E	-0.028	-0.009	0.027	0.049	-0.028	-0.073	-0.075	0.045	-0.001	0.0054	0.0364	-0.03	-0.133	-0.135	-0.044
F	0.0303	-0.074	0.0604	-0.176	0.1092	-0.003	-0.043	-0.027	-0.062	-0.105	-0.047	0.079	-0.024	0.0835	0.027
G	0.1066	0.0747	0.0563	0.0175	-0.05	-0.005	0.0284	0.0615	0.0917	0.1011	0.0042	-0.038	0.057	0.0012	0.0586
H	-0.064	-0.04	0.0323	0.0799	0.0599	0.0316	0.0181	0.0826	-0.142	0.1021	-0.01	0.0211	0.0127	-0.058	-0.023
I	-0.075	-0.074	0.0474	-0.091	0.0098	-0.137	-0.104	-0.076	0.0424	-0.072	-0.027	-0.031	-0.014	-0.045	-0.067
K	-0.028	0.0364	-0.074	-0.142	-0.049	0.0341	-0.055	0.007	0.0477	-0.121	-0.087	-0.078	-0.147	-0.087	0.0421
L	-0.014	-0.071	-0.054	0.0102	-0.028	-0.102	-0.008	-0.227	-0.035	-0.073	-0.139	-0.09	-0.174	-0.036	-0.029
M	-0.034	-0.116	-0.011	-0.012	0.0408	0.0094	0.0327	-0.05	-0.017	0.0486	0.0274	-0.032	0.008	0.0041	0.0405
N	-0.062	0.1062	-0.009	-0.142	0.1303	-0.053	-0.051	0.0328	-0.09	0.0374	-0.039	0.0807	0.061	0.0046	-0.066
P	0.0112	-0.012	0.0505	0.1528	0.0432	0.0645	0.0292	0.0712	0.0515	0.0501	-0.062	0.1056	0.1734	-0.013	-0.007
Q	-0.125	-0.118	-0.153	-0.001	-0.047	0.0411	0.0502	0.0309	-0.002	0.0006	0.0785	0.0425	0.0084	-0.055	-0.011
R	0.0967	-0.003	-0.028	-0.056	-0.054	0.0765	-0.043	0.0832	-0.006	-0.045	0.0471	0.0007	-0.075	0.0117	0.0103
S	0.069	0.0236	0.0173	0.071	0.0195	0.0446	0.1136	0.0838	-0.087	0.0329	0.0255	-0.008	-0.057	0.119	0.0305
T	0.0744	0.0245	-0.047	-0.037	-0.066	-0.015	-0.051	-0.096	0.062	0.0181	0.0152	0.0151	0.0049	-0.05	-0.003
V	-0.134	-0.009	0.0399	-0.044	-0.134	-0.076	-0.067	-0.071	-0.013	-0.021	0.0214	-0.059	-0.002	0.0288	-0.039
W	-0.044	0.0024	0.0738	0.0739	0.0446	-0.07	-0.044	-0.07	0.0314	0.0724	-0.064	-0.058	-0.044	0.0152	-0.044
Y	0.0364	-0.062	0.0265	0.1107	-0.046	-0.057	0.0172	0.0086	0.0262	-0.037	0.036	0.0447	0.085	0.0576	0.0599
X	0	0	0	0	0	0	0	0	0	0	0	0	0	0	0

	-35	-34	-33	-32	-31	-30	-29	-28	-27	-26	-25	-24	-23	-22	-21
A	0.1078	-0.053	0.076	0.0092	0.0807	0.0495	0.0143	-0.014	0.0893	-0.035	-0.126	-0.025	0.1111	0.1006	0.0204
C	-0.104	0.0474	0.0539	-0.06	-0.033	0.0483	0.0293	-0.098	-0.024	5E-05	0.0061	0.005	0.0202	-0.024	0.0224
D	-0.066	0.1112	0.0742	-9E-04	-0.038	0.043	-0.018	-0.003	-0.058	-0.086	0.008	0.0413	-0.101	-0.089	0.0005
E	-0.094	-0.017	-0.105	0.0163	-0.082	-0.09	-0.033	-0.1	-0.081	-0.003	0.0602	-0.003	-0.034	-0.069	0.0262
F	0.0481	-0.029	-0.012	0.0066	-0.15	-0.033	-0.045	-0.045	-0.056	0.0154	-0.053	0.0627	0.0729	-0.05	-0.057
G	0.0749	0.0986	0.0989	0.0212	0.0594	0.0615	0.0604	0.1023	0.0551	-0.009	0.0205	-0.074	0.1262	0.0962	0.0319
H	-0.093	-0.068	-0.046	-0.014	-0.07	0.0626	0.0838	0.0308	-0.009	0.0047	-0.083	-0.065	-0.051	-0.092	-0.034
I	0.0151	-0.091	0.0078	-0.039	-0.087	-0.075	-0.087	-0.067	-0.022	-0.052	-0.037	-0.065	-0.041	-0.092	-0.095
K	0.0217	-0.169	-8E-04	-0.048	0.0654	0.0216	0.0118	-0.081	-0.033	-0.093	0.0112	-0.022	0.02	0.0933	-0.047
L	-0.116	-0.016	-0.004	0.0186	-0.122	-0.101	-0.153	0.0304	-0.03	-0.055	-0.156	-0.086	-0.083	-0.011	0.0281
M	-0.028	0.0162	-0.046	0.1046	0.0607	-0.012	-0.012	-0.02	-0.014	0.0923	0.0545	0.0361	-0.021	0.0722	0.0194
N	0.0405	-0.167	0.0249	0.0357	0.0281	-0.064	-0.099	0.0221	-0.112	0.0407	0.0495	-0.019	0.0288	-0.045	-0.019
P	0.1226	0.06	-0.056	0.0903	0.0624	0.1106	0.0984	0.0531	0.0057	0.0085	0.113	0.1134	0.0433	0.0835	0.0353
Q	-0.042	0.0296	-0.093	-0.004	0.1234	-0.053	-0.02	0.0469	-0.067	0.0633	0.0397	0.0413	-0.021	-0.161	-0.063

R	-0.044	-0.03	-0.011	-0.067	0.0063	0.0464	0.0342	0.0959	0.0084	0.0688	-0.054	0.1095	-0.016	0.0339	0.0898
S	0.0726	0.0425	-0.076	-0.032	-0.008	0.0354	0.0462	-0.023	0.0621	0.038	-0.05	-0.069	-0.042	-0.044	-0.013
T	-0.113	-0.012	-0.006	0.0229	-0.109	-0.174	0.0055	0.0061	0.0057	-0.023	-0.009	-0.127	-0.129	-0.072	-0.099
V	-0.044	0.0705	-0.112	-0.104	-0.097	-0.004	-0.064	-0.07	-0.047	0.0121	0.0326	-0.024	0.0229	-0.05	-0.022
W	0.0221	-0.064	0.0638	-0.082	0.0145	-0.082	-0.024	-0.064	0.0554	-0.03	-0.057	0.0887	-0.044	-0.069	-0.044
Y	-0.011	0.056	0.0884	0.0653	0.0639	0.025	0.0233	0.0257	0.1588	0.0043	0.1184	0.061	-0.101	0.083	0.1046
X	0	0	0	0	0	0	0	0	0	0	0	0	0	0	0
	-20	-19	-18	-17	-16	-15	-14	-13	-12	-11	-10	-9	-8	-7	-6
A	-0.06	-0.035	0.1861	-0.007	0.1043	-0.063	-0.059	0.0479	-0.186	-0.051	0.0184	-0.05	0.0362	0.0451	-0.104
C	0.0167	-0.035	-0.006	-0.031	-0.057	0.0927	-0.053	-0.013	-0.117	-0.092	-0.077	0.0131	-0.026	-0.102	-0.028
D	0.0147	-0.072	-0.128	-0.075	-0.008	-0.018	0.0987	0.0436	-0.003	0.0024	0.0053	0.0301	-0.023	-0.057	-0.087
E	-0.05	0.0024	-0.119	-0.157	-0.036	-0.104	-0.061	-0.037	0.0265	-0.081	-0.072	-0.115	0.0532	-0.098	-0.025
F	-0.019	0.0351	0.0195	-0.086	-0.079	-0.016	0.0227	-0.059	-0.039	-0.043	0.0038	-0.028	-0.002	-0.039	-0.014
G	0.0662	0.0728	0.1093	0.1237	-0.011	0.18	0.1285	0.1093	0.035	0.1348	0.0691	0.0962	0.1135	0.0179	0.073
H	-0.088	0.0165	0.099	0.0182	-0.025	-0.108	-0.117	-0.136	-0.048	-0.059	0.0249	-0.081	-0.013	0.0347	-0.014
I	-0.027	-0.047	-0.121	-0.112	-0.015	-0.197	0.0045	0.0568	-0.02	-0.057	-0.087	-0.07	-0.046	-2E-04	0.0167
K	-0.035	-0.082	-0.087	0.0453	0.0657	-0.052	-0.08	-0.144	-0.017	0.0843	-0.098	-0.124	-0.064	-0.032	-0.086
L	-0.011	-0.055	-0.071	-0.005	-0.208	-0.027	-0.088	0.015	0.0029	-0.04	-0.098	-0.067	-0.171	-0.118	-0.051
M	-0.052	0.0811	-9E-04	0.0741	-0.008	-0.013	-0.059	-0.055	-0.026	-0.153	0.039	0.054	0.0418	-0.068	0.0155
N	0.0677	0.0437	-0.008	-0.064	-0.038	0.0617	-0.036	0.0854	-0.076	0.0257	0.0038	0.0185	-0.022	-0.074	-0.041
P	0.0071	-0.034	0.0421	-0.01	0.0925	-0.046	0.0756	0.0404	0.0355	0.0512	0.1554	0.0216	-0.01	0.0823	0.0351
Q	-0.031	-0.006	0.0388	-0.138	-0.065	0.0142	-0.05	-0.046	0.0014	0.0145	-0.01	-0.001	0.0187	0.0292	-0.054
R	-0.016	0.1334	0.0358	0.1929	0.1293	0.0454	-0.005	-0.029	0.1353	0.0842	0.0697	0.139	0.0666	0.2082	0.0256
S	0.0112	0.0045	-0.002	-0.029	-0.011	-0.014	0.0156	0.0255	0.0269	-0.009	-0.065	0.0875	0.0068	-0.007	0.0441
T	-0.021	-0.039	-0.077	0.0732	-7E-04	-0.029	0.0305	-0.142	0.0432	-0.064	0.0261	-0.027	0.0095	0.0327	0.0517
V	0.0415	-0.095	-0.06	-0.014	0.0076	-0.053	-0.043	-0.021	0.0122	0.0232	-0.255	-0.077	-0.02	0.062	0.0857
W	0.0136	-0.044	-5E-04	-0.075	-0.063	0.0316	-0.032	-0.043	-0.008	-0.069	-0.002	0.0733	-0.057	-0.063	-0.015
Y	0.0799	0.0828	-0.039	0.0198	0.0685	0.0432	0.0341	0.0262	0.0211	-0.005	0.129	-0.034	-0.058	-0.09	0.0434
X	0	0	0	0	0	0	0	0	0	0	0	0	0	0	0
	-5	-4	-3	-2	-1	0	1	2	3	4	5	6	7	8	9
A	0.0533	0.0464	0.068	0.1346	0.0116	0	-0.056	0.1081	-0.002	-0.008	0.0783	0.0383	0.0215	0.07	-0.034
C	-0.112	0.0184	0.005	-0.033	0.0163	0	-0.027	0.0487	-0.078	-0.027	-0.059	-0.027	-2E-04	-0.015	-0.069
D	-0.103	-0.107	-0.093	-0.067	-0.038	0	-0.132	-0.053	-0.048	-0.033	-0.086	-0.01	-0.084	0.0328	0.0044
E	-0.076	-0.143	-0.083	-0.098	-0.093	0	-0.001	-0.07	-0.127	-0.094	-0.174	-0.096	-0.018	-0.193	-0.087
F	0.02	-0.064	-0.032	-0.066	-0.045	0	0.0352	-0.157	0.09	-0.056	-0.157	0.0253	0.0836	0.0643	-0.181
G	0.1241	0.1074	0.1307	0.1699	0.1667	0	0.136	0.1798	0.1209	0.1331	0.1955	0.1302	0.0804	0.1712	0.1339
H	-0.009	0.0526	-0.14	-0.104	-0.053	0	-0.044	-0.005	0.0164	-0.073	-0.02	-0.026	-0.012	-0.131	-0.063
I	-0.044	-0.048	-0.079	0.0028	-0.082	0	-0.156	-0.127	-0.153	-0.024	-0.075	-0.031	-0.068	-0.207	0.0112
K	-0.123	-0.049	-0.022	-0.033	-0.101	0	-0.099	-0.038	-0.011	-0.035	0.012	0.0095	-0.038	0.0298	0.0453
L	-0.103	-0.122	-0.112	-0.032	-0.1	0	-0.139	-0.071	0.0652	-0.017	-0.041	-0.066	-0.021	-0.149	-0.15

M	0.0617	0.0086	0.1509	-0.04	0.0305	0	-0.091	0.0339	0.0366	-0.053	-0.025	0.0217	-4E-04	-0.097	0.0089
N	-0.051	0.0023	-0.146	-0.079	-0.072	0	-0.042	0.0296	-0.124	0.0184	0.0576	-0.017	-0.01	0.0689	0.0443
P	-0.048	0.1158	-0.04	-0.039	0.0924	0	-0.111	-0.041	-0.113	-0.013	0.0521	-0.01	0.1044	0.005	0.0284
Q	-0.048	0.0141	0.0246	-0.089	0.0329	0	-0.173	-0.103	-0.045	0.0147	-0.109	-0.027	0.0215	-0.075	-0.113
R	0.1702	0.163	0.1904	0.1321	0.1322	0	-0.111	0.1139	0.086	0.1565	0.0189	0.0164	0.0215	0.0795	0.0339
S	0.0659	0.0644	0.0775	0.0823	0.0092	0	-0.122	-0.117	0.0158	-0.004	0.0531	0.082	-0.15	0.0103	0.0134
T	0.0085	-0.029	-0.015	-0.201	-0.032	0	-0.105	0.0116	-0.053	-0.041	-0.018	-0.083	-0.024	-0.05	0.0214
V	-0.096	-0.167	-0.052	-0.008	-0.166	0	-0.068	-0.16	-0.008	-0.046	-0.083	-0.112	-0.132	-0.02	-0.018
W	0.0111	-0.086	-0.043	-0.056	-0.036	0	-0.004	-0.05	0.003	-0.033	-0.043	-0.01	0.061	-0.063	0.112
Y	0.0719	-0.023	-0.124	-0.025	-6E-04	0	-0.087	-0.127	0.0099	-0.037	-0.074	-0.012	0.0673	0.009	0.0082
X	0	0	0	0	0	0	0	0	0	0	0	0	0	0	0
	10	11	12	13	14	15	16	17	18	19	20	21	22	23	24
A	-0.071	0.1115	-0.003	-0.048	0.0329	0.0466	-0.057	0.0911	0.0458	-0.035	-0.113	-0.099	-0.047	0.0418	-0.031
C	-0.002	-0.057	-0.062	-0.02	-0.02	0.0036	0.0147	0.0218	-0.022	-0.017	0.0459	-0.019	-0.114	-0.031	-0.087
D	-0.025	-0.059	-0.102	-0.097	-0.103	-0.071	0.0553	-0.028	0.0847	-0.04	-0.034	0.0276	-0.041	-0.009	-0.024
E	-0.042	-0.085	-0.056	-0.085	-0.07	-0.055	-0.135	-0.072	-0.065	-0.077	0.0656	-0.138	-0.09	0.0066	-0.008
F	-0.022	-0.181	-0.035	-0.071	-0.037	-0.087	-0.037	0.0369	-0.041	0.0852	-0.097	-0.003	0.0166	0.0268	0.0503
G	0.1316	0.1305	0.094	0.1973	0.0677	0.0875	0.1138	0.098	0.1374	0.1319	0.1401	0.1314	0.0765	0.1606	0.062
H	0.0089	-0.081	-0.028	0.0317	0.0018	-0.002	-0.007	-0.154	-0.042	-0.062	-0.022	-0.094	0.0422	0.0228	0.0024
I	-0.041	-0.133	-0.133	-0.033	0.0217	0.0015	-0.021	-0.125	-0.067	-0.066	0.0127	-0.134	-0.108	-0.101	-0.078
K	-0.124	0.0406	-0.025	0.054	-0.099	-0.086	0.027	-0.115	-0.06	-0.069	-0.074	0.0001	-0.072	-0.053	-0.139
L	-0.124	-0.037	-0.02	-0.105	-0.032	-0.058	-0.086	0.0051	-0.218	-0.011	-0.127	-0.009	0.0055	-0.089	-0.03
M	-0.024	0.1217	0.114	-0.076	-0.029	0.0593	-0.054	-0.058	-0.011	0.003	-0.146	-0.011	-0.018	0.011	0.0128
N	-0.08	-0.191	-0.026	-0.126	-0.085	-0.033	-0.094	0.0194	0.0013	-0.019	-0.032	-0.037	-0.01	-0.053	0.0173
P	0.0047	0.0537	0.0437	0.0809	0.0774	0.0616	0.1305	-9E-04	0.0815	0.0053	0.0607	0.086	0.1484	0.0387	0.0385
Q	-0.002	0.0036	-0.086	-0.046	0.0017	-0.101	-0.071	0.0434	-0.092	-0.034	0.066	-0.041	-0.021	-0.119	-0.051
R	0.1617	0.0526	0.0887	0.0478	0.107	0.0736	0.131	0.0661	0.0309	0.0742	0.0923	0.0632	0.0706	0.0003	0.0586
S	0.0335	0.0204	0.004	0.0227	-0.071	0.0453	0.0104	-0.047	0.0102	-0.016	0.0251	0.0641	0.0425	0.0187	0.0241
T	0.0386	0.0135	0.0053	-0.004	0.0769	-0.003	-0.169	-0.129	0.0332	-0.029	-0.059	-0.082	-0.014	-0.029	-0.031
V	-0.035	-0.115	-0.042	-0.094	0.0454	0.0254	-0.09	-0.007	-0.143	-0.029	-0.08	-0.071	-0.156	-0.034	0.0297
W	-0.086	0.0116	-0.029	-0.029	-0.081	-0.029	0.0055	-0.001	-0.014	0.0041	-0.044	-9E-04	-0.057	0.0098	-0.051
Y	0.0371	-3E-04	0.0864	-0.012	-0.007	-0.045	-0.003	0.101	0.0933	-0.024	0.0455	0.0537	0.0936	-0.04	0.056
X	0	0	0	0	0	0	0	0	0	0	0	0	0	0	0
	25	26	27	28	29	30	31	32	33	34	35	36	37	38	39
A	0.0352	-0.078	-0.01	0.0785	-0.005	0.0872	0.0384	-0.001	-0.057	-0.071	0.0171	-0.034	-0.108	-0.02	-0.002
C	0.0554	0.0796	-0.119	0.0805	-0.057	-0.082	-0.031	-0.007	-0.019	0.0159	0.0475	-0.006	0.0567	-0.1	0.0045
D	0.0038	0.0169	-0.179	0.0317	0.0325	0.0073	0.0383	-0.04	-0.029	-0.071	-0.034	0.0241	-0.094	0.0458	0.0776
E	-0.165	-0.05	-0.043	-0.05	-0.102	-0.108	-0.041	0.0552	0.0014	0.0567	-0.076	-0.032	0.0044	-0.025	-0.089
F	-0.018	-0.014	0.0345	0.0201	0.0154	0.0095	0.0081	-0.135	-0.021	0.0062	0.0561	-0.032	0.0527	-0.017	-0.032
G	0.0983	0.0876	0.1609	0.1102	0.0018	0.1436	0.0832	0.0861	0.1179	0.1812	0.0919	0.1503	0.118	0.1524	0.1222

H	-0.025	-0.002	0.0158	0.0339	-0.022	0.0024	-0.002	-0.076	0.0116	-0.049	0.022	-0.077	-0.016	0.0342	-0.053
I	0.0023	0.0583	-0.001	-0.058	-0.123	-0.063	-0.033	-0.088	-0.067	-0.047	-0.031	-0.011	-0.067	-0.05	-0.06
K	-0.035	-0.098	-0.052	0.0208	-0.037	-0.012	0.0488	0.02	0.0014	-0.021	-0.04	-0.177	-0.059	-0.278	0.0005
L	-0.143	-0.003	-0.137	-0.048	-0.046	-0.041	0.0168	-0.043	-0.084	0.0451	-0.027	-0.074	-0.122	-0.038	-0.043
M	-0.049	0.0334	-0.001	-0.076	-0.01	-0.052	0.0078	0.0032	0.0489	-0.086	0.0347	0.0063	0.0344	0.0666	0.0617
N	-0.127	-0.114	0.1213	-0.016	0.0157	-0.006	0.042	0.1053	-0.029	0.137	0.0436	0.0749	-0.071	-0.051	-0.041
P	-0.03	0.0799	-0.053	-0.011	0.0087	-0.038	-0.038	-0.139	-0.031	-0.029	0.0767	0.0283	0.1116	0.001	0.0193
Q	-0.044	-0.135	-0.05	-0.089	0.0175	0.0073	0.0275	-0.015	0.043	-0.164	-0.016	-0.011	-0.021	0.0198	-0.075
R	0.0868	0.1023	0.0102	-0.04	0.1475	0.1034	0.0267	-0.001	0.0526	0.024	-0.04	0.0432	0.031	-0.011	0.0008
S	0.1289	-0.006	-0.02	0.0149	0.0251	-0.1	-0.184	-0.05	-0.026	-0.04	-0.009	0.0289	0.0342	0.003	-0.014
T	-0.085	-0.062	0.0113	-0.098	0.0498	0.0047	-0.082	0.0878	-0.04	-0.037	-0.094	0.0436	-0.071	0.0145	-0.024
V	-0.034	-0.095	-0.032	-0.009	0.0036	-0.013	-0.024	0.033	0.0328	-0.077	-0.104	-0.152	-0.124	0.0284	0.0123
W	0.0059	-0.025	0.0537	-0.051	-0.044	-0.044	-0.013	0.0625	-0.064	-0.071	-0.071	-0.037	0.1338	-6E-04	-0.059
Y	0.0528	0.0105	0.0389	-0.01	-0.062	-0.071	0.047	-0.017	0.0353	-0.048	0.0647	0.0104	0.0876	-0.024	0.0157
X	0	0	0	0	0	0	0	0	0	0	0	0	0	0	0

	40	41	42	43	44	45	46	47	48	49	50
A	0.0106	0.0233	-0.019	0.084	0.1432	-0.068	0.1096	0.0323	-0.071	-0.041	-0.066
C	-0.036	-0.042	0.0215	0.0098	-0.1	-0.043	-0.043	-0.058	-0.031	-0.101	-0.024
D	-0.013	0.0012	-0.044	-0.009	-0.106	-0.068	0.0243	0.1201	-0.039	-0.032	-0.022
E	-0.062	-0.113	-0.059	0.0104	-0.038	-0.079	-0.028	-0.14	0.003	-0.12	0.0697
F	-0.018	-0.041	-0.082	-0.049	-0.063	-0.113	0.0422	0.1692	0.08	-0.044	-0.01
G	0.0862	0.1371	0.0749	0.0625	0.0891	0.1088	0.0524	0.082	0.092	0.1086	0.0849
H	-0.096	-0.049	-0.026	-0.012	0.0435	0.0909	-0.073	0.0767	-0.011	-0.01	0.0264
I	0.0308	-0.084	-0.064	0.0306	-0.109	-0.068	-0.066	0.0642	-0.032	-0.126	0.1013
K	-0.06	-0.165	-0.009	-0.057	0.0002	-0.006	-0.073	-0.032	-0.007	0.1088	-0.041
L	-0.006	0.0158	-0.062	-0.053	-0.041	-0.047	0.0436	-0.095	0.0243	0.0155	-0.039
M	-0.041	0.0521	0.0387	-0.171	-0.096	0.0433	0.0608	-0.001	0	0.0153	0.0016
N	-0.06	0.0714	0.1664	0.0256	0.0675	0.0523	0.0646	-0.137	-0.028	-0.077	-0.046
P	0.0083	-0.041	-0.019	-0.02	0.0071	0.1576	-0.061	-0.057	-0.033	0.0143	-0.015
Q	0.0315	0.0017	0.0231	-0.013	-0.075	-0.076	0.0047	-0.009	0.0414	0.0046	-0.053
R	0.0779	0.0008	-0.003	0.0354	0.0071	0.0034	-0.024	-0.015	0.1119	0.0769	-0.125
S	0.0954	0.0114	-0.014	-0.019	0.0457	0.0022	-0.059	-0.011	-0.089	-0.022	-0.063
T	-0.002	-0.019	-0.029	0.001	0.0046	-0.107	-0.083	0.0317	-0.043	0.0177	0.0584
V	-0.109	-0.053	-0.038	-0.093	-0.082	-0.068	-0.003	-0.065	-0.052	-0.031	-0.09
W	-0.052	0.006	-0.052	0.0964	0.0471	-0.095	-0.001	-0.059	-0.045	0.092	-0.006
Y	-0.02	0.0869	0.0682	0.0616	0.0341	0.0788	0.0058	-0.064	0.0197	-0.078	0.1623
X	0	0	0	0	0	0	0	0	0	0	0

Lysine methylation.

	-35	-34	-33	-32	-31	-30	-29	-28	-27	-26	-25	-24	-23	-22	-21
A	-0.056	0.0901	0.0416	-0.042	0.1104	-0.002	0.0385	-0.031	0.041	0.1175	0.1305	0.0171	0.0327	0.059	0.0726

C	-0.076	-0.049	-0.058	-0.096	-0.044	-0.09	0.0482	0.0225	-0.015	0.0435	-0.075	-0.03	0.0534	-0.014	-0.121
D	-0.024	-0.111	-0.006	0.0097	0.0765	0.0219	-0.051	0.0017	0.0541	0.0423	0.0058	-0.02	-0.029	-0.01	-0.146
E	-0.037	-0.005	0.0011	0.0397	0.1176	-0.053	-0.01	0.0297	0.0313	-0.084	0.0268	-0.041	-0.064	0.0237	0.0277
F	-0.083	-0.198	-0.167	-0.006	-0.109	-0.166	0.0548	-0.102	0.0622	0.0546	0.0196	0.0479	-0.108	-0.041	0.0981
G	0.0515	0.0759	-0.007	0.0047	0.0774	-0.009	0.0265	-0.065	0.063	-0.067	0.0338	0.1144	-0.072	0.0096	0.0556
H	-0.014	0.0656	0.1095	0.0303	-0.062	0.0393	0.118	0.0435	-0.104	-0.019	-0.028	-0.074	0.0209	0.08	-0.001
I	-0.15	-0.025	0.0187	-0.087	-0.066	-0.022	0.1064	0.0083	-0.06	0.143	0.0869	0.0411	0.1631	-9E-04	-0.088
K	0.0832	0.0006	0.0663	0.0052	0.0585	0.0124	-0.095	0.0213	-0.049	-0.018	-0.122	-0.155	0.0112	-0.007	-0.013
L	-0.082	-0.045	-0.008	-0.046	0.0002	0.0866	-0.049	-0.077	-0.095	-0.01	-0.133	-0.071	-7E-04	0.0486	-0.017
M	0.097	0.0499	0.0081	0.0281	0.0207	0.0319	-0.024	0.0029	-0.066	0.0164	0.003	-0.049	0.0568	-0.023	-0.053
N	-0.005	0.0607	0.0193	0.0747	-0.016	-0.037	-0.07	-0.061	0.0376	0.0181	0.0751	-0.041	-0.097	0.0754	0.0519
P	0.0118	-0.076	0.0124	-0.046	-0.098	-0.031	-0.069	-0.077	0.0263	-0.014	-0.04	0.0872	-0.085	-0.032	0.027
Q	-0.033	-0.009	-0.091	-7E-04	-0.21	0.0125	0.0275	-0.01	-0.055	-0.106	-0.037	0.0579	-0.1	-0.223	-0.051
R	-0.005	0.0741	-0.017	-0.005	-0.092	0.0041	-0.187	0.1591	0.0279	0.009	-0.029	-0.015	-9E-04	0.0397	0.0097
S	0.097	-0.016	0.0146	0.1111	-0.042	0.0438	-0.013	-0.016	0.0292	-0.105	0.051	-0.07	-0.012	0.0202	0.0638
T	0.0118	0.0104	-0.06	0.0646	-0.065	0.0643	0.096	-0.006	-0.08	-0.115	-0.023	0.0755	0.0658	-0.074	0.0005
V	0.097	-0.019	0.0634	-0.159	-0.043	-0.016	0.0144	0.0681	-0.049	-0.063	0.051	-0.088	-0.032	-0.017	-0.129
W	-0.034	-0.065	-0.053	0.008	0.0077	-0.069	0.02	0.0018	0.0267	-0.046	-0.052	0.0561	0.0801	0.0078	0.0802
Y	-0.034	-0.058	-0.127	-0.058	-0.032	-0.04	0.0078	-0.045	0.061	0.0823	-0.08	0.1526	0.0568	-0.074	-0.039
X	0	0	0	0	0	0	0	0	0	0	0	0	0	0	0
	-20	-19	-18	-17	-16	-15	-14	-13	-12	-11	-10	-9	-8	-7	-6
A	-0.034	-0.004	-2E-04	-0.026	0.0903	-0.004	-0.003	-0.037	-0.061	0.0381	0.0561	0.0532	0.0925	-0.032	-0.013
C	-0.019	0.044	-0.015	0.0218	-0.035	0.0977	0.0616	-0.041	0.0304	0.0166	-0.074	-0.074	0.0018	0.1132	-0.047
D	-0.019	-0.071	0.0118	-0.052	-0.004	-0.075	-0.028	-0.127	0.0039	0.0239	-0.068	-0.065	-0.13	0.0855	0.0172
E	-0.132	-0.047	-0.045	0.0057	-0.078	-0.09	-0.011	-0.016	-0.046	0.0354	-0.052	-0.061	-0.129	0.0236	0.0533
F	-0.108	0.0678	0.0892	-0.101	0.0342	-0.064	0.0597	-0.111	0.0008	-0.156	-0.02	-0.036	0.0009	-0.111	-0.115
G	0.0031	-0.03	0.0146	0.0484	0.0264	-0.061	-0.046	0.0013	0.0209	-0.042	0.1039	0.0831	-0.033	-0.013	0.0238
H	0.0346	0.003	-0.036	0.0286	0.0241	-0.054	-0.078	0.0714	0.0218	-0.072	0.0116	-0.036	-0.015	-0.094	-0.06
I	0.0264	-0.073	-0.057	0.0263	0.0649	0.0218	-0.025	0.0303	0.0777	-0.095	-0.106	-0.07	0.0048	-0.073	0.0459
K	0.0878	0.1404	-0.031	0.0961	0.0006	0.0322	-0.119	0.0954	0.1265	0.1398	-0.001	0.0838	0.1374	0.1161	0.1614
L	0.0176	0.1025	-0.004	-0.009	-0.006	-0.041	-0.14	0.0174	-0.079	-0.099	-0.104	-0.011	-0.011	-0.162	-0.066
M	0.0767	-0.057	0.0563	0.0642	0.0286	-0.058	-0.003	0.1031	0.0089	0.0559	0.0296	0.036	0.0144	-0.016	0.003
N	0.0157	-0.057	0.0375	-0.024	-0.048	-0.039	0.0028	-0.006	-0.026	0.0133	-0.042	0.0317	0.0009	-0.01	0.086
P	0.0597	-0.065	0.0144	-0.05	-0.077	0.0357	0.0299	0.0133	-0.114	0.0325	0.0317	-0.119	-0.1	-0.087	-0.032
Q	-0.063	-0.045	0.0276	0.0008	0.095	-0.062	0.0271	-0.055	-0.105	-0.077	0.1419	-0.043	0.0027	-0.021	-0.141
R	-0.164	-0.008	-0.054	-0.04	0.0176	-0.046	0.0145	-0.033	0.1346	0.0301	-0.055	0.0132	0.0267	0.011	-0.014
S	0.0728	0.0844	0.0267	0.0602	-0.043	0.0672	0.0358	-0.072	-0.042	-0.053	0.0368	0.0362	0.0512	0.0085	-0.063
T	0.0332	-0.06	0.0372	-0.139	-0.006	0.0542	0.064	0.0397	-0.022	-0.056	0.0015	-0.029	-0.084	0.0846	-0.021
V	0.0127	-0.108	-0.082	-0.058	-0.125	0.0861	0.0901	-0.009	-0.108	0.0679	0.0288	0.0638	0.0918	-0.029	0.0486
W	-0.084	-0.069	-0.034	0.0405	-0.025	-0.069	0.0608	0.1886	-0.069	-0.046	-0.069	0.0107	-0.007	-0.01	-0.039

Y	0.0442	0.025	0.0433	-0.005	0.0184	0.1639	0.0293	-0.067	0.0841	-0.004	-0.084	-0.002	0.0144	0.0709	-0.015
X	0	0	0	0	0	0	0	0	0	0	0	0	0	0	0
	-5	-4	-3	-2	-1	0	1	2	3	4	5	6	7	8	9
A	-0.033	-0.059	-0.053	0.0239	-0.07	0	-0.017	-0.01	-0.028	0.1205	0.0328	-0.073	0.0171	-0.036	0.0267
C	-0.037	-0.098	0.047	-0.058	-0.025	0	-0.002	-0.084	-0.035	-0.084	-0.025	-0.029	-0.044	-0.024	-0.084
D	0.0234	0.059	-0.055	-0.054	0.0185	0	0.0087	0.0489	-0.017	0.0786	0.0616	-0.054	0.0798	-0.104	0.0536
E	-0.009	-0.069	-0.035	-0.027	-0.11	0	-0.012	-0.16	-0.025	-0.016	-0.059	-0.087	0.0108	0.0379	0.0122
F	-0.058	-0.061	-0.078	0.0122	0.1085	0	-0.035	0.0367	0.0065	-0.095	0.0309	0.1329	-0.091	-0.022	-0.113
G	-0.091	0.0899	0.0172	0.0573	0.0194	0	-0.017	0.0505	0.1033	-0.053	0.1086	-0.023	-0.042	0.0872	0.0003
H	-0.052	0.0204	-0.019	-0.071	-0.107	0	-0.107	0.0248	0.0414	0.0614	-0.015	-0.083	-0.014	-0.053	-0.018
I	0.0978	0.0608	0.0291	-0.036	-0.088	0	0.018	0.0304	0.1069	0.0079	-0.084	0.0536	-0.118	0.0373	0.0434
K	0.1121	0.0013	0.0809	0.1275	0.1828	0	-0.103	-0.114	-0.032	0.0221	0.0032	0.0112	0.0641	0.1362	-0.053
L	-0.083	-0.045	0.0063	-0.156	-0.015	0	-0.034	0.03	-0.049	-0.095	-0.102	-0.095	-0.09	-0.141	-0.006
M	-0.12	0.0431	0.0603	0.0412	-0.03	0	-0.033	-0.024	0.0469	-0.01	-0.023	-0.049	-0.098	0.0895	0.0235
N	0.0391	0.056	0.0365	0.0665	-0.133	0	-0.015	-0.075	0.0057	-0.026	-0.015	0.0386	0.1174	-0.01	-0.063
P	0.0144	0.0728	0.0237	0.0665	0.0549	0	0.0816	0.0426	-0.039	0.0893	-0.037	0.0065	0.1078	-0.012	0.0105
Q	-0.051	0.0208	0.0176	-0.018	-0.13	0	-0.041	-0.162	-0.159	-0.108	-0.035	-0.104	-0.001	-0.095	-0.126
R	-0.014	-0.019	0.0115	-0.073	0.1836	0	0.085	-0.024	-0.036	0.0302	0.008	-0.034	0.0627	-0.003	0.0283
S	-0.047	-0.021	-0.006	0.063	0.0266	0	0.1338	0.0347	0.0566	-0.077	0.0312	-0.008	-0.01	0.0292	-0.008
T	0.0321	-0.064	0.0206	-0.004	-0.033	0	-0.039	0.0713	-0.026	-0.129	-0.022	0.1288	-0.11	0.0105	0.0033
V	0.0098	0.0134	-0.046	0.0092	-0.032	0	0.0464	0.0529	-0.056	-0.003	0.0106	0.1267	-0.056	-0.046	0.1021
W	0.0898	-0.002	-0.074	-0.079	-0.01	0	0.0459	0.0852	0.035	0.0355	0.015	-0.084	-0.003	-0.014	-0.008
Y	0.1093	-0.061	0.0101	0.0268	-0.028	0	-0.117	0.0367	0.0476	0.0988	0.0394	0.0548	0.0359	0.0022	0.0359
X	0	0	0	0	0	0	0	0	0	0	0	0	0	0	0
	10	11	12	13	14	15	16	17	18	19	20	21	22	23	24
A	0.0775	0.1368	-0.009	0.0019	-0.046	-0.071	-0.114	-0.028	-0.16	-0.056	0.0139	-0.015	0.0178	-0.065	0.0933
C	-0.074	0.0147	-1E-04	0.048	-0.085	-0.069	-0.009	0.0103	-0.039	0.0357	-0.019	-0.064	0.0323	-0.11	0.034
D	-0.038	0.1302	-0.09	-0.141	0.0574	0.051	-0.119	-0.024	0.09	-0.025	-0.04	0.1197	-0.071	0.0485	0.0254
E	-0.051	-0.12	0.0098	-0.132	0.0362	0.0016	-0.063	-0.091	0.0295	-0.167	0.0139	-0.025	-0.06	-0.022	-0.059
F	0.0446	0.0264	0.0074	0.0272	-0.106	-0.026	-0.026	-0.01	0.0507	-0.008	-0.035	0.0338	-0.006	0.0387	-0.12
G	0.0193	-0.074	0.0746	-0.063	-0.031	0.0226	-0.013	-0.043	-0.111	0.0668	0.0228	0.0296	0.0129	0.0615	-0.106
H	0.0358	-0.157	-0.075	0.0096	-0.106	-0.134	0.0196	0.0294	0.031	0.0491	-0.054	-0.109	0.0512	-0.008	0.1035
I	0.0528	-0.042	-0.183	-0.051	0.0977	0.0114	0.0097	0.0264	0.0198	0.0632	0.0519	0.0185	-0.061	0.0851	-0.112
K	-0.058	0.0903	0.1246	0.0456	0.1155	0.1197	0.1244	0.0897	0.119	0.0684	0.0084	0.0696	0.0176	0.0073	0.0416
L	-0.029	0.0214	-0.103	0.0589	-0.118	0.0154	0.008	-0.055	0.0303	-0.191	-0.01	-0.051	-0.021	-0.064	0.0103
M	-0.006	-0.049	0.0443	0.0356	-0.051	-0.059	-0.009	0.0596	0.0166	-0.012	-0.057	-0.075	0.0467	-3E-05	-0.048
N	-0.012	0.0191	0.0292	0.0263	-0.081	0.076	-0.154	-0.04	-0.035	-0.001	-0.076	0.0179	0.0726	0.0373	0.0434
P	0.1075	0.0448	-0.002	0.005	0.0231	-0.036	0.1139	0.0093	0.0399	0.0033	-0.051	0.0506	0.0368	-0.093	-0.124
Q	-0.064	-0.134	-0.015	0.0069	-0.05	-0.021	-0.048	-8E-04	-0.091	-0.075	-0.015	-0.114	-0.03	-0.087	0.0561
R	-0.154	-0.096	0.0245	0.1345	-0.063	-0.032	0.0734	0.0025	0.0343	0.0954	0.112	-0.029	-0.056	-0.12	0.0604

S	0.0429	-0.006	-0.086	-0.142	-0.097	-0.08	-0.001	-0.01	0.018	0.0588	-0.017	0.0249	0.0248	-0.003	0.0139
T	-0.009	-0.024	0.0557	0.0283	0.073	0.0067	0.0134	0.1216	-0.075	-0.048	0.0376	-0.06	-0.019	0.0526	0.0594
V	-0.018	-0.04	0.0245	-0.008	0.0635	0.0019	0.0029	-0.023	-0.148	-0.079	-0.057	-0.038	0.0687	0.1124	-0.115
W	-0.008	0.0089	-0.052	-0.04	0.0155	-0.019	0.0154	0.0554	-0.04	0.1792	0.0163	0.0631	0.0098	-0.047	-0.064
Y	0.0275	0.0105	-0.015	0.1136	0.1053	0.0363	0.0116	-0.091	-0.012	-0.012	0.0698	-0.043	-0.087	0.0179	0.0058
X	0	0	0	0	0	0	0	0	0	0	0	0	0	0	0

	25	26	27	28	29	30	31	32	33	34	35
A	-0.046	-0.014	0.0582	-0.132	0.0003	0.0615	0.0044	0.0809	0.0223	0.0694	0.0397
C	-0.028	-0.078	-0.028	-0.081	-0.106	-0.007	0.0085	-0.028	0.1018	-0.041	-0.011
D	0.0828	0.0096	0.0089	-0.023	0.0962	0.096	-0.068	-0.198	-0.093	-0.143	0.0643
E	-0.078	-0.054	-0.02	-0.089	0.0701	0.071	-0.005	0.0357	-0.02	-0.058	-0.053
F	0.0759	0.0313	-0.055	0.0728	-0.038	0.0089	0.0601	0.043	-0.138	0.0061	0.1038
G	0.0803	-0.087	0.0241	0.019	-0.044	0.0274	0.0306	0.0119	0.0357	0.0317	-0.032
H	0.0421	-0.012	0.0835	0.0683	-0.106	0.0064	0.0154	0.0046	0.0875	0.0555	-0.041
I	0.0051	-0.084	0.036	0.0943	-0.119	-0.151	-0.05	0.0218	-0.002	-0.061	-0.042
K	0.0195	-0.023	0.1249	0.1031	-0.075	0.0341	-0.043	0.0357	0.038	0.092	0.0198
L	0.016	0.0846	-0.049	-0.047	-7E-04	-0.001	0.0212	-0.028	-0.23	0.0145	-0.02
M	-0.029	-0.061	-0.045	0.0102	0.0541	0.0383	0.0339	0.0125	-0.022	-0.069	-0.008
N	-0.102	-0.018	0.0289	-0.021	0.0245	-0.034	0.0045	-0.085	0.0241	0.0176	0.0093
P	0.0313	-0.004	-0.05	0.0194	-0.047	0.0542	-0.074	0.0638	-0.068	-0.089	-0.004
Q	-0.096	0.0564	-0.172	-0.049	-0.084	-0.074	-0.091	-0.086	0.0084	0.032	-0.08
R	0.0159	0.021	-0.076	0.029	-0.135	-0.134	0.0001	0.023	0.0349	0.091	-0.016
S	0.0234	-0.008	0.056	0.0037	0.1129	-0.15	0.036	0.0644	-0.038	-0.089	0.0107
T	-0.035	0.0048	-0.038	0.0383	0.0861	0.071	0.0654	-0.02	0.0208	-0.038	-0.135
V	-0.041	0.1339	-0.054	-0.03	0.0933	-0.057	0.0118	-0.038	0.0834	0.057	0.0926
W	-0.059	-0.04	-0.001	0.0109	-0.001	-0.053	-0.028	-0.012	-0.005	-0.077	0.0131
Y	0.0392	-0.016	0.0242	-0.053	0.024	-0.029	0.011	-0.142	0.0956	0.0015	0.0236
X	0	0	0	0	0	0	0	0	0	0	0
